# What Will We Do? The Action Plan From a Brazilian Professional Football Club Youth Academy Facing the COVID-19 Pandemic

**DOI:** 10.3389/fspor.2021.589459

**Published:** 2021-06-07

**Authors:** Murilo dos Reis Morbi, Annie Rangel Kopanakis, Pau Mateu, Billy Graeff, Renato Francisco Rodrigues Marques

**Affiliations:** ^1^School of Physical Education and Sport of Ribeirão Preto, University of São Paulo, Ribeirão Preto, Brazil; ^2^Pontifical Catholic University of Campinas, Campinas, Brazil; ^3^Institut Nacional d'Educació Física de Catalunya, Universitat de Barcelona, Barcelona, Spain; ^4^Federal University of Rio Grande, Rio Grande, Brazil

**Keywords:** football, COVID-19, action plan, youth academy, sport career, learning cultures, social inclusion

## Abstract

In 2020, the world was affected by the COVID-19 pandemic, which remains a major challenge for most countries today. In Brazil, football clubs' youth academies have faced a disruption of their regular activities. In order to study how the learning cultures of a Brazilian professional football club youth academy have been changed, and the alternatives created by the club's staff within this context, this perspective article aims to analyze how they have structured the Under-15 (U15) team learning culture during social isolation due to the COVID-19 pandemic. Through document and thematic analysis on a Brazilian professional football club's youth academy program, we promoted a dialogue between the process of adaptation to remote theoretical-tactical teaching with the learning theory proposed by Hodkinson and collaborators. The main theme of analysis of this study was the remote structure of the theoretical-tactical learning and physical training. Challenged with the need to transpose face-to-face activities into a learning culture based on remote communication, the U15 team coaching staff created a process to prescribe physical training, and to teach and discuss football tactical issues with young players during the period of social isolation. This perspective article shows that it is possible for sports institutions to create programs for the development of young athletes within the social isolation/distancing context, considering both theoretical-tactical learning and physical training processes. The adaptation to remote environments as structures for the learning culture seems a challenge, but is also a good alternative for young players to develop their interpretation and perception of football theoretical-tactical issues.

## Introduction

Since the beginning of 2020, the world has been facing a pandemic caused by COVID-19 (Del Rio and Malani, 2020). In April, 19, 2021, 140.8 million cases and 3 million virus-related deaths have been reported globally (WHO, 2021). As COVID-19 has caused impacts in various fields of society such as health, economics, education, and sport, it can be said that this pandemic represents an intersection between the natural and social dimensions that surround human life (Badiou, 2020). To face this scenario, quarantine and social distancing have been adopted as strategies to prevent the spread of the virus in several countries (Frahsa et al., 2020; Wilder-Smith and Freedman, 2020), as any meetings, festivals, professional, or youth football games offer a genuine contamination risk (McCloskey et al., 2020).

Worldwide, several sport activities have been impacted due to measures aimed at preventing people agglomerations. Major sports leagues have been disrupted and the Tokyo Olympics have been postponed to 2021 (IOC, 2020), as countries around the world are implementing measures to contain the virus spreading (McCloskey et al., 2020; Pons et al., 2020). Evans et al. (2020) proposed questions about the impact of the pandemic in the sports context, in order to reflect on how the lives of institutions, athletes and other sport participants will be transformed and what new considerations may guide the practice of teaching and training in the future. In addition, the authors sought to elucidate how the pandemic revealed the inequalities of access to and consumption of sport in society, bringing to light the need for social sciences studies on the sport world facing COVID-19, considering cultural, social, and political contexts.

Within the sports scenario, football youth academies consist in very relevant social spaces where young players commonly migrate to invest in a sport career, being away from their families and homes. The joining of a football club youth academy allows young athletes to be immersed in a very specific football culture (Darby et al., 2007; Esson, 2015), reinforcing their dream of developing an elite football career (Bourke, 2003; Agergaard and Ungruhe, 2016; van der Meij and Darby, 2017). A better understanding on how football youth academies work can offer information about the management processes of these institutions, and how they provide protection and guarantee the rights of young players (Yilmaz et al., 2020).

In Brazil, football is a relevant sport, interesting to young players for several reasons, including the belief about the possibility of social mobility (Rial, 2008; Damo, 2014; Marques et al., 2020). Because of this, Brazilian laws regulate the football clubs' youth academies system (Brazil, 2011), which encourage these institutions to be certified by the Brazilian Football Confederation (CBF) when they meet the minimum requirements to offer adequate care to young players. Some of the conditions for obtaining this certification are (a) to ensure that young players are registered for and attending school, and (b) to guarantee health and psychological care for players and their families. When the clubs are certified, they legally acquire the right of priority when signing the first professional contract with the young players.

In Brazilian youth football, competitions are organized according to a state/regional sport federations' management system. These events are divided into several age groups: Under-11 (U11), U13, U15, U17, and U20. In the state of São Paulo, the Paulista Football Federation (FPF) approved the return of professional competitions in July, 2020 (FPF, 2020a). However, most of their youth competitions remained suspended with no date for return, except for the São Paulo Championship U20, which restarted at the end of October, 2020 (FPF, 2020b). Within this context, the eventual return of activities depends on the epidemiological situation in the region in which the competitions take place (São Paulo, 2020). Consequently, efforts to better understand the effects of the COVID-19 pandemic in the sports field (Giulianotti and Collison, 2020; Mateu and Marques, 2020; Bandyopadhyay, 2021), and especially in grassroots sport (Frahsa et al., 2020; Kelly et al., 2020; Pons et al., 2020) are very relevant.

In the specific case of Brazil, even before the pandemic, the country was already facing political and economic crises that mainly affected the poorest socioeconomic groups. As little is known about the characteristics of COVID-19 transmission in a context of great social inequality (Barreto et al., 2020), the conditions of social vulnerability of a significant part of Brazilian population (Graeff et al., 2019) suggest that dealing with this new disease is a highly complex challenge (Buheji et al., 2020; Mateu and Marques, 2020). In this context, Brazilian federal and state governments have adopted several measures to reduce the spread of COVID-19, which include restrictions on the functioning of schools, universities, places of coexistence, public transportation, and other venues that facilitate the agglomeration of people, such as related to social and sporting events (Oliveira et al., 2020). As a result, the suspension of the in-person activities in youth football academies obligated Brazilian clubs to adapt the ways of training, teaching and learning to remote communication alternatives. Considering that young football players' learning and career development are long term processes, consisting mainly of practical experiences (Barker et al., 2014; Barker-Ruchti and Schubring, 2016), the social isolation related to the COVID-19 pandemic led to structural and relevant modifications on football youth academies' learning cultures.

The concept of learning cultures does not address geographical places (related only to concrete sites), but social and symbolic spaces with imprecise and overlapping boundaries, where people interact, constructing learning processes through interpersonal relations (Hodkinson et al., 2007b, 2008). Although this concept was created based on in-person learning relations, it is also applicable to the remote learning condition, because it also involves similar structures of social relationships among participants. Besides that, learning cultures are constituted by power relations within the culture of a given institution (in this case, a Brazilian professional football club youth academy), its ways of organization, social interaction among club staff and young players, and their dispositions for action (Lee and Price, 2016).

In order to explore and discuss how the learning cultures of a Brazilian professional football club youth academy have been changed and the alternatives created by the club's staff within this context, this perspective article aims to analyze how they have structured the U15 team's learning culture during social isolation due to the COVID-19 pandemic.

To justify this analysis, we believe that a better understanding of a Brazilian professional football club youth academy's remote learning culture, created during social isolation because of the COVID-19 pandemic, can contribute to the body of knowledge and practical actions on several areas, such as sport management, sport pedagogy, sociology of education, sociology of sport, among others, both for immediate and for future interventions.

## Methods

The investigated Brazilian professional football club youth academy participates in state level championships and has the ‘Formative Club Certificate', a quality seal endorsed by the CBF for clubs that offer good conditions for developing young players. The club consists of 125 players aged between 13 and 20 years old, distributed among groups ranging from the U15 to U20. The investigated group (U15) consists of 27 players. In addition, there is a Department of Human Development (DHD) in this institution that offers psychological and social care for players.

After the interruption of training and competitions due to the COVID-19 pandemic, and the returning of young athletes to their family homes (around 50% of players' families live in different towns than the club's headquarters), the club adjusted its procedures and ways of interaction with athletes to remote communication alternatives. At that time, several activities of psychological support from the DHD were proposed for athletes and their families, considering the new social context. In addition to this, the club's coaching staff, together with the head manager of the youth academy, proposed diversified physical and game activities for players training at home.

Within the scenario of sports activities interruption, in the following we present and analyze the learning culture structure of this Brazilian professional football club youth academy U15 team, located in a countryside city in the state of São Paulo. For this, we took as main sources of data the action plan for remote activities created by the club's coaching staff (details about this document are presented in the Supplementary Material). To accomplish this task, a documentary analysis method was initially performed (Bowen, 2009; Smith and Sparkes, 2014). Then, data analysis was performed based on the Thematic Analysis method (Braun and Clarke, 2006, 2012, 2019), adopting the following interactive and dynamic procedures (Braun and Clarke, 2019): (a) the researchers became familiar with the data, reading and rereading the plans and reports provided by the club; (b) a first coding proposal based on learning cultures was created from a sociocultural perspective (Hodkinson et al., 2007a, 2008); (c) the data were reviewed and grouped into three main themes; (d) production of results' report.

**Figure 1:**
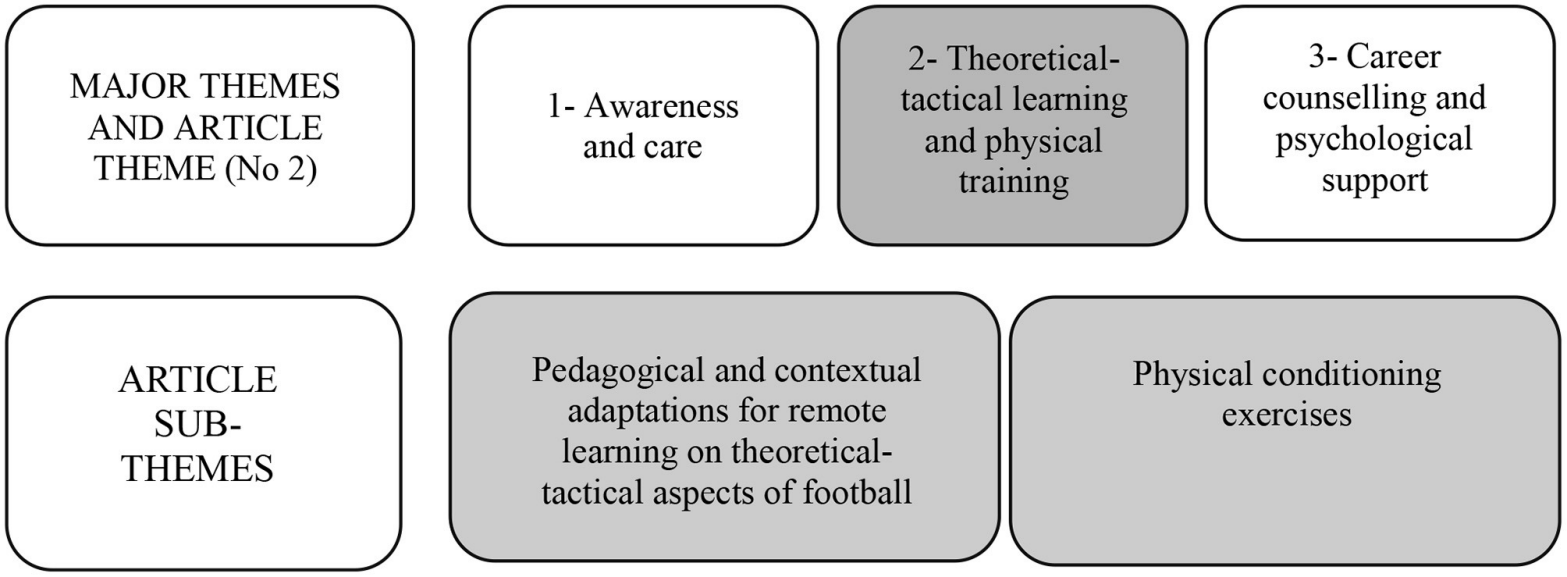
Map of the data analysis process.

The article's themes were composed by some analytical categories, such as: (a) remote tactical learning activities; (b) physical conditioning exercises; (c) retrospective analysis on the players' trajectories before joining the club. The three major themes are: (1) Awareness and care; (2) Theoretical-tactical learning and physical training; (3) Career counseling and psychological support (Figure 1). Although the construction of the thematic map addresses the three themes, the authors' option for this article was to discuss and present the analysis only from the theme “Theoretical-tactical learning and physical training” and its sub-themes “Pedagogical and contextual adaptations for remote learning on theoretical-tactical aspects of football” and “Physical conditioning exercises.” The choice to name it as “theoretical-tactical” is because the situation of teaching and learning was based on a remote interaction, in which it was not possible for young players to practice football, causing those contents to be approached only on a conceptual and theoretical dimension.

## Results and Discussion

As the purpose of this article is to analyze how a Brazilian professional football club youth academy staff have structured its U15 team's learning culture during social isolation due to the COVID-19 pandemic, the authors' choice was to present and discuss the action plan related to the Theoretical-tactical learning and physical training.

The U15's action plan and reports provided by the club's coach staff concerns ways to enable the continuity of the teaching specific football topics during COVID-19 pandemic. This document is part of the Training Curriculum of the youth academy, and aims to structure a remote learning culture considering both the social isolation conditions and the young players' sociocultural background, creating specific and particular horizons for learning (Peim and Hodkinson, 2007; Hodkinson et al., 2008) in the pandemic context. Considering that the learning of young players is structured by a dialectical relation between social context and the individual disposition to learn (Hodkinson et al., 2008; Barker-Ruchti et al., 2016), the learning cultures based on the collective construction of knowledge and the sharing of personal perceptions can create an important sense of coexistence and favorable conditions for learning (Hodkinson et al., 2007b), especially during a period of social distancing/isolation (Kelly et al., 2020).

### Pedagogical and Contextual Adaptations for Remote Learning on Theoretical-Tactical Aspects of Football

One of the actions of the analyzed professional football club youth academy was to provide access to digital means for remote communication between young players and the club's coach staff. As a first step, a group was created on an instant messaging platform, whereby young players could interact with their teammates, coach staff, and the DHD. This creation of a remote space altered the learning culture shared by these social agents. Therefore, one aspect highlighted in the club's “Training Curriculum” is to enable an environment in which young players can express their own understandings about theoretical-tactical issues. Although in-person and face-to-face training sessions are not possible during the pandemic social isolation, the remote learning culture contributed to young players present and discuss their ideas about football theoretical-tactical issues with teammates and coaching staff. Furthermore, considering that each interaction among agents provides transformations on the learning cultures (Hodkinson et al., 2008; Barker-Ruchti et al., 2016), it is also possible that the learning processes have changed and became different during the young players interactions with digital media.

Adding to this, before the U15 coach staff started the remote activities with young players, the DHD did adjustments on the activities to be proposed, according to the socioeconomic and sociocultural conditions that young players have had in their homes. In line with concerns regarding not only football development, but also the athletes' living conditions and guarantee of basic rights, the club supported athletes who were in a situation of social vulnerability. This work involved the specialized attention of psychological and social service cares from the DHD, being close to the young athletes that demanded special attention (such as anxiety situations and financial or social distresses due to the context of pandemic and its limitations on conviviality). In this sense, multidisciplinary work among educators, managers, coaches, physiotherapists, psychologists, and social assistants was fundamental for the creation of a remote learning culture that could offer institutional support and suitable remote learning conditions.

The support from the DHD was decisive for the coach staff planning the theoretical-tactical learning activities. Not all young players have had good access to the internet and to technological devices. Therefore, activities that depended on the players watching long lasting football game videos for theoretical-tactical reflection and collective discussion were not easily available for every player. Taking this information into account, some activities were adapted by the coach staff. As an example, young players had to read the information in the text messages shared into the group, and based on this they should draw, explain, and produce texts about the theoretical-tactical issues that have been trained before the pandemic. The main concern on this activities' adaptation was to vary the teaching and learning methods, in addition to diversifying the materials for the fulfillment of tasks, seeking to reduce the inequality of internet access among young players.

Within this process, activities proposed for the theoretical-tactical learning were based on pre-pandemic lived situations during training and matches sessions. Based on videos and literature sent by coach staff, players studied and answered questionnaires related to tactical issues. Then, in remote meetings among players and coaches, theoretical tactical topics were discussed and analyzed in-depth (the Supplementary Material presents examples of activities and their aims).

### Physical Conditioning Exercises

Brazil is a country in a socioeconomic development process with a high level of social inequality (Graeff et al., 2019). It produces a scenario where several young players have difficulties of access to means of remote communication, creating some barriers for the engagement with physical exercise and maintenance of fitness in social isolation/distancing situation. While some social groups may devote more time to maintaining their physical fitness and conditioning, using better materials, exercises, and nutrition, others do not have the same opportunities (Mateu and Marques, 2020). For those young players who did not have the structural or contextual conditions to perform the planned training at home, as the difficulty of accessing information through technological devices because of social vulnerability and specific familial dynamics, there was an alternative plan which was based on exercises that could be done in a more limited space and with a lack of material resources (such as squats, push-ups, burpees, and resisted exercises utilizing body weight).

## Preparing For the Future: Conclusions, Learning Culture Practical Implications, and the Youth Academy Future in the Post-Pandemic Scenario

The aim of this article was to analyze how a Brazilian professional football club youth academy staff have structured the U15 team learning culture during social isolation due to the COVID-19 pandemic. According to this, we presented how interventions and interactions between the club's staff and young players were planned and adapted into a specific learning culture during the social distancing period.

Faced with the need to transpose in-person activities into remote communication, the club's coaching staff involved with the U15 team was obligated to plan and create a favorable environment to social interaction among all athletes. The work developed with the U15 football players through interactive meetings with coaches, and also the DHD interventions, offered an in-depth understanding about what were the conditions that each player was living at their homes during pandemic social isolation. In parallel to the perspective of learning as a cultural practice in the sports context, it is evident that the social structure offered by the club was strongly related to the practices performed before the pandemic. The remote activities sought to reconstruct the club's learning culture, but still shared a very close interaction among players and coach staff.

Important problems caused by the COVID-19 pandemic are related to the social isolation, and consequently the transformation of the learning culture of this club from in-person relations to a remote environment. This seems to be one of the biggest challenges imposed over sports institutions during the pandemic context. However, such factors may present themselves as an opportunity for clubs to reflect on their teaching/training practices and investigate socioeconomic and sociocultural conditions lived by young players. A better understanding of contextual factors can help with the adaptation of teaching/training and support the expansion of the remote learning cultures possibilities by sport institutions.

This perspective article shows that it is possible for sports clubs to create programs for the development of young athletes within the social isolation/distancing context, considering both theoretical-tactical learning and physical training processes. The adaptation to remote environments as structures for the learning culture seems a challenge, but is also a good alternative for young players developing their interpretation and perception of football theoretical-tactical issues. The possibility of creating a remote environment meets the need for social distancing imposed by the COVID-19, besides favoring new technologies that can be used as a teaching tool even in a post-pandemic world.

The contribution of this work is specially related to the presentation of an adaptation process from a Brazilian professional football club youth academy to the conditions imposed by social distancing due the COVID-19 pandemic. However, it can also be considered as a first step for future studies on applications of remote activities to young players' training programs. It seems relevant that future research should focus on exploring the perspective of the social agents involved in sport programs, with special attention to young players.

## Data Availability Statement

The original contributions presented in the study are included in the article/Supplementary Material, further inquiries can be directed to the corresponding author.

## Author Contributions

MM, AK, and RM: study design. BG and PM: critical friend. MM, AK, PM, BG, and RM: manuscript preparation. All authors contributed to the article and approved the submitted version.

## Conflict of Interest

MM and AK are staff members of the club that was analysed in this case report. MM is coach of U15 team and AK is chief psychologist of the Department of Human Development. The remaining authors declare that the research was conducted in the absence of any commercial or financial relationships that could be construed as a potential conflict of interest.
