# Supplementary material for: What Will We Do? The Action Plan From a Brazilian Professional Football Club Youth Academy Facing the COVID-19 Pandemic
Source: Front Sports Act Living. 2021 Jun 7;3:589459. doi: 10.3389/fspor.2021.589459 (PMC8215199; doi:10.3389/fspor.2021.589459)
Supplement: Supplementary file 1 [file Data_Sheet_1.PDF]

Activity Report U15

Action plan during covid-19 social isolation

Activity 1 - Club A 3 x 0 Club B match analysis

Objective: To analyse collective tactical actions.

Method: Video analysis and answering questions.

1-How many shots on goal did OUR team in the game? What about the opponent?

2-What game system did the opponent use in the offensive actions? What about the defensive ones?

3-Describe our best collective offensive action during the game.

4-Describe our best collective defensive action during the game.

5-What didn't we do well on the offensive actions? What about the defensive ones? What should we improve?

Activity 2 - Material about collective actions into the game - Construction, Creation and Finishing

Objective: To study the sent PDF material; to analyse and interpret supporting videos that show actions related to the different phases of the game (construction; creation and finishing); To create a draw for each game phase with possible collective tactical actions.

Method: Pdf document and video analysis; drawing collective tactical actions.

Activity 3 - Study material on Back/Winger relationship and intersectors play

Objective: To study the sent pdf material; to analyse and interpret past game videos; to answer an objective questionnaire.

Method: Pdf document, video analysis and objective questionnaire.

Activity 4 - Scoring actions video analysis

Objective: To watch and to analyse ten videos of scoring actions and describe what collective tactical action was decisive for it.

Method: Video analysis and description of collective tactical actions.

Activity 5 – Interpretation of the specific concepts used during training and match sessions

Objective: To stimulate the interpretation of specific concepts and slangs present in training and match sessions.

Method: Reading of the list with concepts and slangs and description about their understanding.

1- "Attracts":

2- "Attacking":

3- "Funnel! Funnel":

4- "Opposite side":

5- "Connection":

6- "Drag":

7- "No contact":

8- "Go inside":

Activity 6 - Understanding different tactical roles into the game

Objective: To stimulate reflection on the playing tactical role position and identify their specific characteristics.

Method: To answer the following questions:

1- Write the main characteristics of the position YOU play. Write the position roles and at least five characteristics of it (if you play in more than one, choose the one you think perform best).

2- Choose a professional player who plays in the same position as you and write what he excels and what he doesn't do so well.
3- What are YOUR main tactical characteristics? Write at least three.
4- What training activity do you think helps in the improvement of your main characteristics?
5- Draw a training activity that can help you in what you need to improve.
6- What do you think you have to improve to be a better player?

Activity 7 - Video on the personal pathway until joining to the club

Objective: To know each young player's life history and stimulate the ability to verbalizing about own life.

Method: To record a video.

Activity 8 - Self-analysis as a football player

Objective: To stimulate reflection and self-evaluation of football performance

Method: To answer an opened questionnaire.

Activity 9 – To study pdf material and answer the questions about technical and tactical issues

Objective: To stimulate reflection about technical and tactical issues.

Method: To study pdf material and answer the questions.

What are the basic techniques of football?

Give examples of attacking individual tactics.

Give examples of defending individual tactics.

What technique do you perform well? Which one do you have to improve?

What football game concepts are related to collective tactics?

Give examples of attacking collective tactics.

Give examples of defending collective tactics

Activity 10 - Video analysis of game actions.

Objective: To analyse individual and collective actions.

Method: Video analysis and answering questions.

1- What moments of the game do the video show? (Offensive action, defensive transition, defensive phase or offensive transition)

2- What game actions can we see in the video?

3- Highlight an individual action of some player.

4- Highlight some collective action that could have been better.

Activity 11 - Studying pdf materials and answering an online questionnaire

Objective: To reflect about tactical issues.

Method: Study support pdf material and answer online questionnaire.

Questionnaire applied:

1) **Mark the alternative that corresponds to the guidelines of "GAME CONSTRUCTION (1)"** *

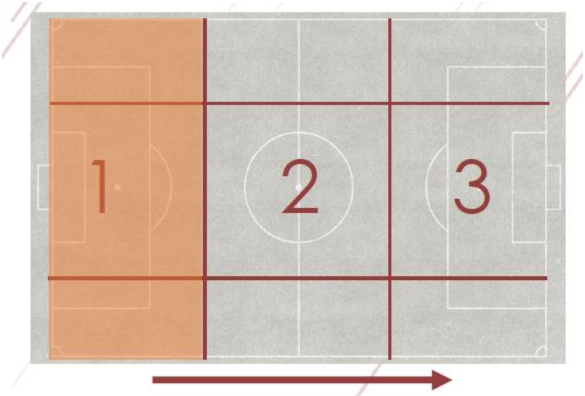

( )"Long pass" / "Diagonal pass"
( ) "Finishing to goal!" / "Support"
( ) "Contactless play" / "Triangle"
( ) "Go ahead" (1vs1) / "Two vs one" (2vs1)

2) Mark the alternatives that match actions related to collective tactics: *

( ) "Drag"
( ) "Turning in the triangle"
( ) "1x1"
( ) "3rd man"

3) Mark the alternative that represents the actions of the "BETWEEN-LINES GAME" *

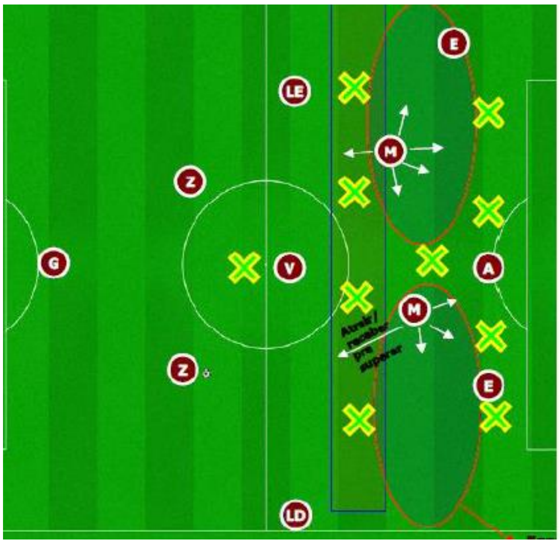

( ) Receive the ball / looking for a pass to forward player
( ) Run to attack space
( ) Receive and make 1vs1
( ) Move to be unmarked.

4) Sign the alternatives that correspond to the actions of "GAME CREATION (2)" *

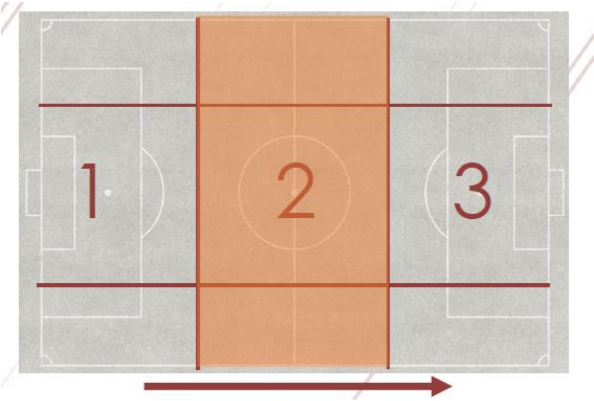

( ) Run with the ball to progress
( ) Use 3rd man to progress on the playing field
( ) "Finishing on goal!" / "Support"
( ) Search for quick passes

Activity 12 - Reflecting on the role position and answering questions

Objective: To reflect on key issues of the player's role position.

Method: Video analysis and answering questions.

1- What individual actions do you think are the most important in our game style/strategy?

2- What collective tactical actions do you see in the video?

4- Do you think that the way we play resembles that of a professional football team? Why?

Activity 13 - Drawing game actions

Objective: To stimulate the ability to understand tactical actions.

Method: Drawing game actions from the names we use in the day-to-day training.

1- 3rd man.

2- Running line.

3- Funnel protection.

4- Pressing and coverage.
